# Peer group social media interactions within a blended learning space

**DOI:** 10.1042/ETLS20253030

**Published:** 2026-01-21

**Authors:** David P. Smith, Sophie M. Pearce, Iosif Giechos, Melissa M. Lacey

**Affiliations:** School of Biosciences and Chemistry, Sheffield Hallam University, Sheffield, S1 1WB, U.K.

**Keywords:** digital learning spaces, group formation, peer-to-peer interactions, social media, student engagement

## Abstract

This study explores how undergraduate students form and engage in peer-to-peer social media interactions within a blended learning environment. Drawing on questionnaire responses from 158 students and focus group data from 12 participants in the School of Biosciences and Chemistry at Sheffield Hallam University, we examine the platforms used, the nature of interactions, and the impact on student experience. WhatsApp, Snapchat and Instagram emerged as the most frequently used platforms, with students primarily discussing coursework, revision and module content. Social media groups were typically formed during face-to-face sessions early in the academic year, highlighting the importance of physical spaces in initiating digital networks. Analysis revealed a dynamic interplay between large cohort-wide groups and smaller, trusted peer groups, each serving distinct academic and social functions. Through focus groups, students reported increased motivation, improved attendance and enhanced learning through these interactions. However, those excluded from early group formation faced barriers to engagement and support. We utilised the ‘Forming, Storming, Norming, Performance’ framework to describe the evolution and impact of these digital peer networks. The findings underscore the need for educators to facilitate early group formation and ensure accessible and clear guidance to prevent misinformation spreading through groups. Practical recommendations are provided to support inclusive and effective digital learning spaces.

## Background

Our previous work shows students sit with peer groups in lecture theatres and form lab partnerships with peers who have similar attainment and background characteristics [1,2]. It is typical for students to bridge physical and digital spaces using social media, with these groups predominantly being closed groups that academics cannot access [3-5]. Absence or exclusion from these spaces, and the subsequent inability to help direct students' learning, has resulted in misconceptions and responses being propagated within peer groups [5,6]. The use of the blended digital learning space for incoming and current students is a grey area for many, with academics forced to make assumptions on how the students are interacting. Here, we aim to better understand how students form and use supporting digital peer group networks in a blended learning space.

## Methods

### Participants

The study ran in the 2022–23 academic year. Students from the School of Biosciences and Chemistry at Sheffield Hallam University were invited to complete online questionnaires during taught sessions within skills modules. The questionnaire was presented to students using two QR (quick response) codes, the first linking to the questionnaire and the second to the participants' information form. To ensure all students were informed about the study, students were also notified about the task by email. Students were recruited across all years of undergraduate study, including associated foundation years.

Students were invited to take part in focus groups by email. Focus groups were facilitated by a student researcher to reduce perceived power imbalances between the focus group attendees and the facilitator. To ensure participants were confident that their focus group responses would remain anonymous, the participant information form stated:

*“The session will be audio recorded for later analysis following written transcription. The study may quote things that are said in the focus group and any quotes will remain anonymous to protect your confidentiality. The interviewer will be the only person to carry out the focus group and listen to the audio recordings and check the written transcriptions to maintain confidentiality”.*

### Data analysis

Online questionnaire data were transferred to Excel, and quantitative analysis was undertaken.

Focus group data were analysed thematically using Braun and Clarke’s [7] six-phase framework in NVivo [7]. Researchers familiarised themselves with the transcripts, systematically coded key phrases and grouped these into themes aligned with the research questions. Themes were reviewed, refined and supported with participant quotes. Note: tutorial groups were 8–12 students within the same year and course of study.

### Ethics

The project gained ethical approval within Sheffield Hallam University’s ethics framework (ER5796357). Explicit consent was gained from participants at the start of the questionnaire and focus groups, and no personal data were collected. General data protection regulation (GDPR) and data management were integrated into the project from its initial design.

## Results

The questionnaire was completed by 158 participants (from 733 invited, 22% uptake) across the School of Biosciences and Chemistry: 7% of students were in their foundation year, 54% in their first year, 23% in their second year and 16% in their final year of undergraduate study. Due to the small participant numbers from several years of study, the data were pooled to increase statistical power. To determine which social media platforms students are using, and the frequency of use, they were asked, *“In general how often do you use the following social media networks to interact with other students in your subject area”*. The most used platforms were WhatsApp, Snapchat and Instagram. A total of 89% of students were using one or more of these top three at least weekly. Students report through focus groups that the choice of platform used depends on the task at hand, with applications like WhatsApp being used in a more formal manner for academic purposes, rather than social media like Instagram, which was used for personal reasons.

*“I use Instagram on more like a personal level to keep up with what’s going on outside of uni, whereas WhatsApp’s just more focused on my course”.*

*“…we usually use WhatsApp within my course, but with my friends student mates its usually Snapchat or Instagram”*

### Social media is used for a range of teaching, learning and assessment-based interactions

**Figure 1:**
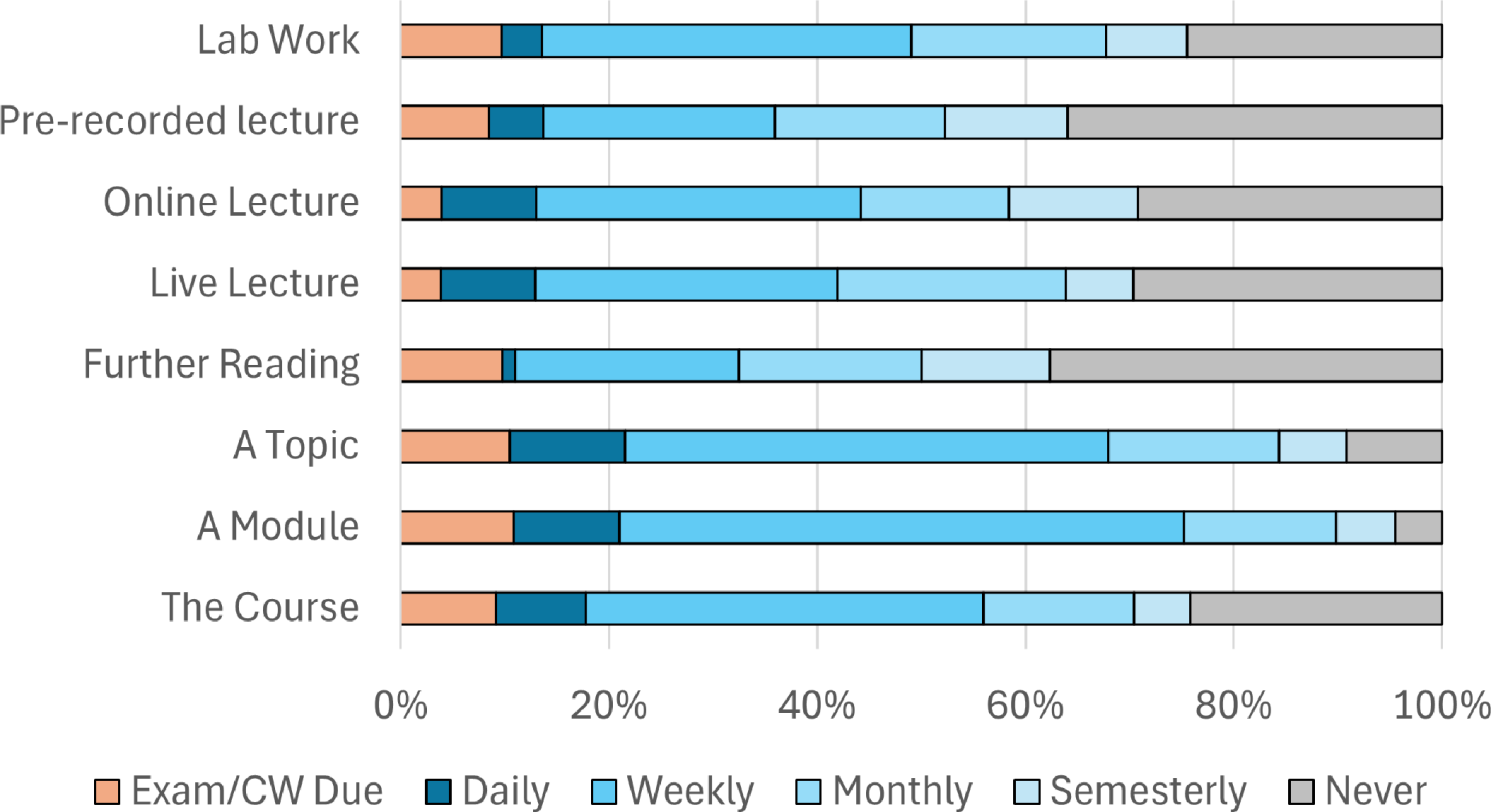
How often students use social media around different aspects of learning (*n*=157).

To determine how students are using social media linked to their learning, within the questionnaire they were asked, *“Thinking about learning, when you are using social media how often is the interaction based around…”* (Figure 1).

Students are using social media to interact with peers to most frequently discuss a specific topic or module, with 61% and 64% of participants, respectively, interacting weekly or daily. Further reading and recorded lectures were the least often discussed, with 37% and 35% of students, respectively, reporting they never discuss these topics.

**Figure 2:**
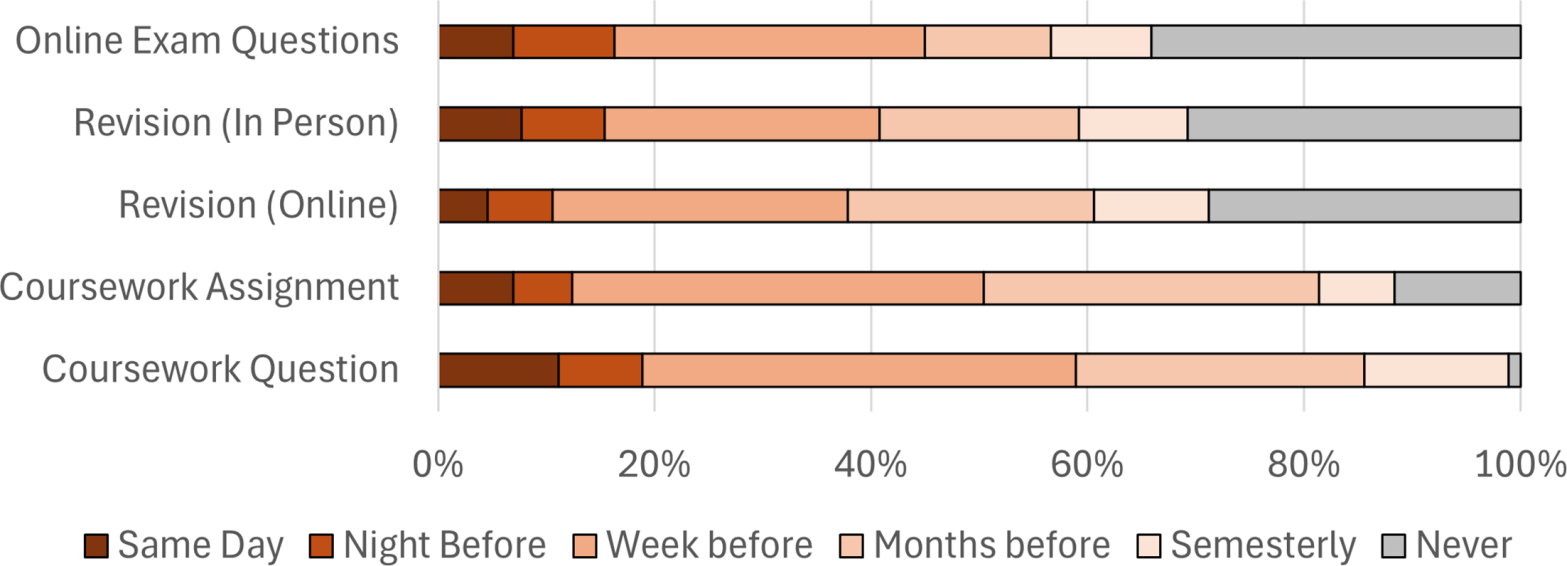
How students use social media peer groups around different aspects of assessment (*n*=154).

To better understand how students are using social media-based peer groups to discuss assessments, participants were asked, *“Thinking about assessments (courseworks & exams), when you are using social media how often is the interaction based around…”* (Figure 2).

Coursework is most frequently discussed by participants in online spaces, with 99% of participants reporting that they interact on social media around coursework questions at least once a semester. Social media peer groups are also used to discuss revision, with 61% and 58% of students discussing revision online or in person (respectively) at least one week before the exam.

### Social media peer interactions have a positive impact on student experience

To determine the perceived impact of social media-based peer-to-peer interactions on students’ learning and attendance, participants were asked, *“Thinking about learning, when you interact on social media with your peers, how often do you find that it encourages you to learn, attend lectures, labs etc”*. A total of 23% of participants stated that social media groups encouraged them with their learning daily or hourly, 41% stated social media groups encouraged them weekly, 11% monthly and 8% semesterly or around exams, whereas 17% of students reported they were never encouraged by these groups.

In addition, students were asked how much they agreed or disagreed with the statement *“Interacting with your peers enhances the University experience?”* on a 5-point Likert scale. The response was overwhelmingly positive, with 84% of students strongly agreeing or agreeing that interacting with peers enhances their university experience, with only 6% disagreeing or strongly disagreeing.

### Social media group formation occurs early and in face-to-face sessions

**Figure 3:**
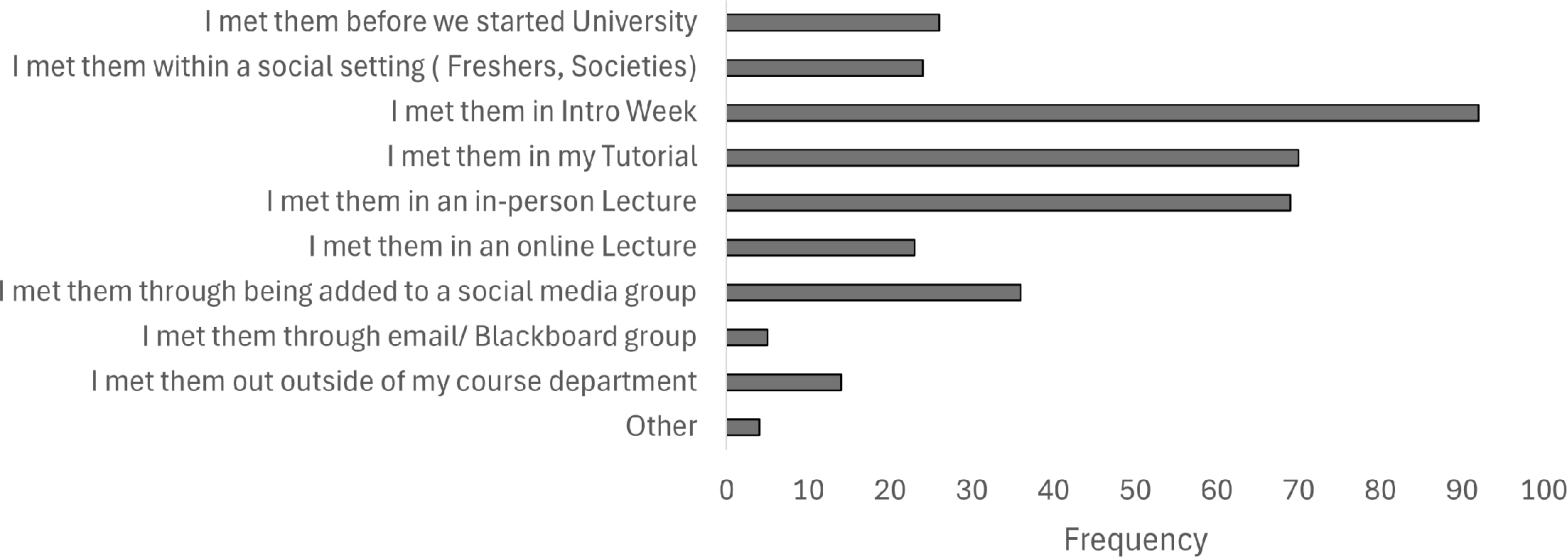
How students initially connect with peers with whom they interact the most on social media. Within the context of a social setting, ‘Freshers’ is a series of Students’ Union events held in the first several weeks of the first semester, specifically aimed at first-year students. ‘Intro Week’ refers to introductory sessions organised by the academics to welcome students to the school and prepare them for the first week of teaching. Tutorials are 8–12 students, whereas lectures are 30–250 students, depending on the course and module. Blackboard is the institutional online learning environment, one function of which is facilitating students’ group work. Note that frequency is more than the total number of participants, as more than one factor could be selected by each student (*n*=157).

To better understand how students form social media peer relationships, they were asked, *“Thinking about your initial meeting, how did you meet your peers that you connect with on social media?”* (Figure 3).

The most frequent methods participants connected initially with peers they interact with on social media were *“I met them in Intro Week”*, followed by *“I met them in my tutorial”* and *“I met them in an in-person lecture”*. This shows the value of interactions in a physical space to support the formation of digital peer group spaces, as well as the hidden value of students' attendance at a range of in-person sessions.

**Figure 4:**
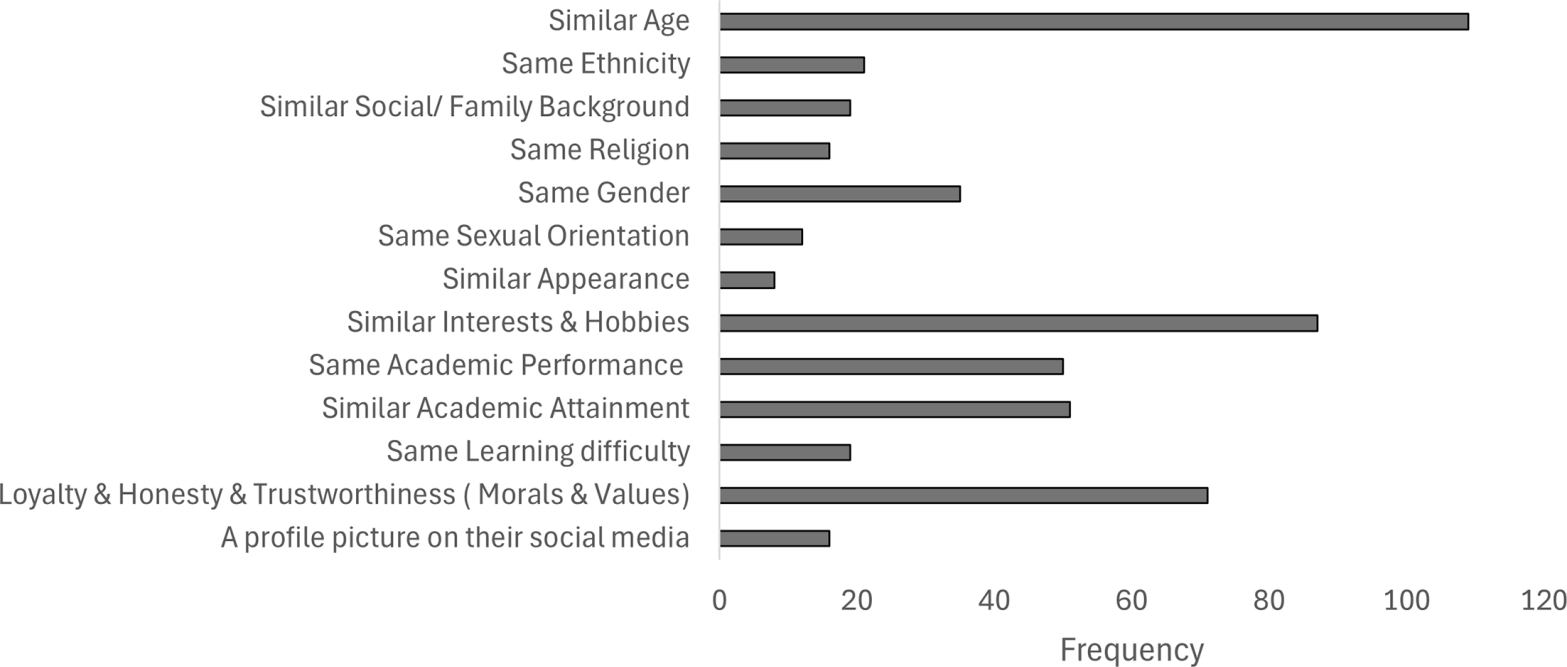
Factors influencing students’ initial social media group formation. Note that frequency is more than the total number of participants, as more than one factor could be selected by each student (*n*=186).

Our previous work on the formation of lab groups showed that group formation of high-achieving students is based around similar levels of engagement, whereas lower achieving groups form around a shared social background [1]. To determine if similar factors were considered in online peer group formation, students were asked, *“Thinking about forming your initial interactions on social media, who are you most likely to form a social media group/interact with? Someone of...”* (Figure 4).

Group formation is primarily driven by age and interest as well as loyalty, honesty and trustworthiness. It is of note that 93% of the School of Biosciences and Chemistry’s newly enrolled undergraduate and foundation students are 21 years old and under. Perceived academic performance and attainment are also significant factors in group formation, mirroring results seen in our previous work [1].

To determine the longevity of the social media groups formed upon starting university, participants were asked*, “Of all the group chats that you were added to and joined at the beginning of the year, do you still use them?”*. A total of 19% of students used groups created at the start of their university studies hourly or daily, 32% used them weekly, 16% used them monthly to semesterly and 14% used them only around exams or coursework deadlines, whereas 19% of students no longer used them (*n*=156).

### Social media group formation is dynamic throughout students’ time at university

To gain a deeper understanding of the central research question of how students form and use supporting digital peer group networks in a blended learning space, three focus groups were conducted (total focus group participants, *n*=12). Focus group questions were informed by the analysis of the questionnaire data and ran for 30–45 minutes and were scaffolded with the following semi-structured open questions.

Q1: In what way do you use social media to help with your learning?

Q2: In what way do you use social media in your social life (student community)? Does this differ from the ways you use social media to help with your learning? If so, how?

Q3: In what way do you use social media to discuss assessment?

Q4: Thinking about your past experiences of using social media to discuss assessment:

- Tell me about a positive experience you have had?
- Tell me about a negative experience you have had?

Q5: In an ideal world, how would you use social media to discuss assessment?

Transcripts were generated and thematically analysed and grouped into the following themes Table 1. Themes gave depth to both the group structures and the way the students used them.

**Table 1:** Core themes identified from focus groups and their occurrences within participant discussions. Focus group participants (*n*=12)

Core focus group theme	Theme occurrence
Forming**:** group formation methods	45
Forming: digital platform choice	69
Storming: discussions based on content vs. general approach	56
Norming: small group vs. large group dynamics	78
Performing: consolidation and reassurance	44

### Group dynamics analysis: Forming, Storming, Norming and Performing

The ‘Forming, Storming, Norming and Performing’ [8] framework was applied as a lens to gain insights into the group dynamics through the students' learning journey.

**Forming:** at the start of the learning journey, large online group chats (often encompassing an entire course or cohort) are primarily used for broad organisation and information sharing and act as the entry point to the wider community. Students observe larger groups as a place for general organisation, for example, where a lecture is and when a deadline is. They provide reassurance and multiple points of view.

*“When we first started for the BMS [Biomedical Science] group we had the links shared in emails and then we just circulated it around whoever’s got whose number”*

*“I also use social media asking people when deadlines are due…”*

Students report that these big groups are invaluable for finding out logistical details, for example, confirming lecture times or locations, clarifying when deadlines are due or sharing timetable changes [9].

*“I think for my group it was the larger group was useful for setting up study groups or meeting up to catch up on content it was certainly useful for if we had a difficult piece of coursework”*

*“I would say the positives, it’s good for organisation…”*

With many participants, large groups can quickly crowdsource solutions to common problems (like finding a difficult classroom or fixing an IT issue) and provide moral support. Knowing that other students share the same concern or have the same question can normalise challenges and encourage help-seeking.

*“So someone will initially make the group chat so for Human Biology I made it and then I put on Facebook, like, this group, oh who’s doing Human Biology this year, first year and then people, kind of, going, oh me and I’ll be, like, oh I’ll add you to this group then and then that’s how it, sort of, starts, through that”.*

Students also discussed that big social media groups can be intimidating, leading to the storming stage.

**Storming:** posting in a large group of perhaps 100–200 people can be daunting for some students. Not everyone feels comfortable broadcasting their questions or confusion to the entire cohort.

*“...obviously you don’t want to put it on the big group chat if everyone’s talking about something else so it’s just a bit easier to have, smaller groups”.*

Once formed, groups are not static, with new, smaller groups forming from big groups and students falling back on the security of larger groups at various points in the student journey. Large groups allow making individual contacts with peers.

*“…it’s quite easy to find other people especially if you don’t have the Snapchat, you can find them from the main group chat where everyone is in it, so you can just quickly add them into a smaller group chat”*

*“We have on the shared WhatsApp group we have a shared digital calendar … and they’ve input all the deadlines for the assessments and course work etc so we get notified”*

Research on peer support groups notes that more introverted students or those anxious about judgment tend to be hesitant to actively participate in large chats [10]. As one student in a focus group explained, it’s *“not as daunting”* to ask questions in a small ten-person chat than in a forum with nearly two hundred peers. This hesitation can lead some members to become ‘lurkers’ who read information in the big group but rarely post. This leads to fragmentation.

*“I want to say the smaller Snap Chat group which is used with our tutorial group … it’s not as daunting to put it in there because there’s ten of us in comparison to 194”*

*“…it’s quite easy to find other people especially if you don’t have the Snapchat or their account you can find them from the main group chat where everyone is in it, so you can just quickly add them into a smaller group chat and it’s quite easy to make your own group anyway”.*

**Norming:** in contrast with the all-in cohort chats, small peer groups (such as private WhatsApp groups among friends or tutorial group chats) offer a more focused setting for learning. Students discuss learning challenges in greater depth and how to approach assessments within these groups. The smaller groups are then more likely places to arrange study meetings and are a place of reassurance with trusted peers.

*“we’ve got, a smaller group between ourselves, a friendship group, and we just use that to talk”*

*“…Arranging to meet up in a library over WhatsApp to revise together… It was nice that we weren’t sort of studying alone”*

These groups, often formed by students who meet through classes or the large cohort chat, serve as safe spaces to explore more deeply into coursework challenges or provide mutual support.

*“I think as well that it’s also really useful to just confirm that you are doing it correctly if you have any doubts of your method or whatever yes”.*

Familiarity lowers the barriers to participation, making it a safer space to share questions and concerns. These groups reinforce belonging, establish shared academic goals and provide a foundation for social and educational well-being.

*“it’s a lot more active, the smaller one, than the bigger one”*

*“There’s a specific question like a maths question or something that and I feel like a lot of people don’t understand that question, so you can then discuss it together in the group chat and you can see what the possible way to get to it is”.*

**Performing**: once group norms are established, students move into effective collaboration and mutual support.

*“…when we do have an assessment due we, all come together to, help each other”*

*“it’s also really useful to just confirm that you are doing it correctly if you have any doubts of your method”*

Peer groups motivate attendance, facilitate joint studying and create accountability for coursework completion. They act as both an academic safety net and a source of social connection. Peer groups do affect motivation and attendance. Having a group of friends or study partners creates a sense of accountability and encouragement to participate in coursework [11]. Students find that peer groups increase their motivation to attend face-to-face sessions, as they have someone to sit with. They are also motivated to complete work; for example, seeing peers engage with coursework can motivate students to be at the same point.

*“…that communication is helping me get myself into Uni, get my attendance up but then also if I am struggling with, like, topics, we’ll text and be, like, let’s go to the library, we’ll watch the lectures, kind of thing and then go over it…”*

*“if I am struggling with, like, topics, we’ll text and be, like, let’s go to the library, we’ll watch the lectures, kind of thing and then go over it…”*

This can be a double-edged sword, with internal validation for practices such as working close to the deadline. When peer influence creates a productive climate, students don’t want to let their friends down in group projects, and they feel motivated to keep pace when they observe others making progress. However, this effect can be negative. With a motivated group, collectively boost each member’s productivity, whereas if the prevailing norm in a group is that everyone procrastinates until a deadline is near, then the group might reinforce poor academic practices.

## Discussion

The mixed methods data presented here show that peer-to-peer social media interactions play a crucial role in enhancing the academic experience of undergraduate students within a blended learning environment. Findings indicate that platforms like WhatsApp, Snapchat and Instagram facilitate effective communication among students, primarily focused on coursework and academic support.

Seen through the Forming, Storming, Norming and Performing model, digital peer groups among students follow a clear developmental trajectory. Large cohort groups mark the forming stage, providing initial connection and organisation. Intimidation in large groups reflects storming, as students negotiate roles and seek comfort from friends. The shift into smaller groups signals norming, with shared trust and routines. Finally, when groups enhance motivation, attendance and academic progress, they reach performing. This framing highlights how digital peer groups evolve from loose collectives into highly functional support systems that significantly shape student learning and belonging.

Educators have a role to play in actively promoting and guiding the establishment of these digital networks to enhance student motivation and learning outcomes while minimising potential misinformation within them.

### Limitations

The study has several limitations that should be acknowledged:

Sample size and diversity: Although 158 questionnaire responses were collected, the sample may not fully represent the diverse student population within the School of Biosciences and Chemistry. Factors such as year of study, demographic background and prior experiences with social media could influence engagement and interaction patterns. The findings may be specific to the School of Biosciences and Chemistry at Sheffield Hallam University and may not be generalisable to other disciplines or institutions.

Focus group limitations: The focus groups consisted of only 12 participants, which may not capture a broad range of perspectives. Smaller focus groups might overlook the voices of students who are less likely to speak up or have differing experiences regarding social media use and peer interactions. The reliance on self-reported data through questionnaires and focus groups can introduce biases, as students may provide socially desirable responses or may not fully recall their experiences accurately.

Temporal context: The study was conducted during a specific academic year (2022–23), and results may vary in different academic years due to changes in curriculum, social media trends or student demographics. The rapidly evolving landscape of social media platforms may affect the relevance of findings over time. However, the general principles should hold.

### Practical recommendations

1. Students benefit from being in social media groups. Allow students time in face-to-face sessions at the start of their student journey to form social media peer groups, especially large, whole cohort groups.
2. Students who are absent for the first weeks of teaching may not have been invited to large social media groups. Evidence presented shows the value of social media groups in supporting students, and thus, students who are not initially included are not able to gain the benefits. Thus, identifying these students who joined courses late or who had limited attendance in the first weeks and encouraging them to seek out course-level social media groups will support their inclusion in the digital learning space.
3. Students are turning to each other to answer assessment-based questions if answers cannot be easily found in teaching materials. To support students in accessing correct information, as opposed to potential misinformation via peers, ensure key information and materials are easily accessible via well-organised virtual learning environments.
4. As with physical spaces, within digital spaces students are at risk of encountering misconduct, harassment, bullying or abuse. Although this was not found within this study, all students need to be aware of their university’s expectations of their behaviour in all settings, as well as how students can report and be supported if they observe or are the victim of misconduct, harassment, bullying or abuse.

## Data Availability

10.6084/m9.figshare.30161449

## Open Access

This article has been published open access under our Subscribe to Open programme, made possible through the support of our subscribing institutions, learn more here: https://portlandpress.com/pages/open_access_options_and_prices#conditional
